# Electronic Properties of Common Psychostimulants in Aqueous Environments

**DOI:** 10.3390/molecules31203625

**Published:** 2026-10-12

**Authors:** Roman Boča, Beáta Vranovičová, Juraj Štofko, Cyril Rajnák

**Affiliations:** 1 Faculty of Health Sciences, University of SS Cyril and Methodius in Trnava, 91701 Trnava, Slovakia; juraj.stofko@ucm.sk; 2 Faculty of Natural Sciences, University of SS Cyril and Methodius in Trnava, 91701 Trnava, Slovakia; beata.vranovicova@ucm.sk

**Keywords:** psychostimulants, quantum chemical calculations, redox properties

## Abstract

We employed advanced quantum chemical methods to investigate the electronic properties of five widely consumed psychostimulants: nicotine, cotinine, caffeine, theophylline, and theobromine. Molecular geometries were optimized in an aqueous environment using density functional theory (DFT) with the B3LYP exchange-correlation functional and an extensive basis set. The calculated structures showed excellent agreement with experimentally determined solid-state structures deposited in the Cambridge Crystallographic Data Centre (CCDC). Full vibrational analyses enabled the determination of thermodynamic properties, including Gibbs energies. For the neutral ground states, we calculated dipole and quadrupole moments, dipole polarizabilities, zero-point vibrational energies, and entropic contributions. Adiabatic ionization energies and electron affinities were obtained from optimized cationic and anionic geometries and used to derive molecular electronegativities, chemical hardnesses, and electrophilicity indices. Reaction Gibbs energies for ionization and reduction were converted into absolute oxidation and reduction potentials. To improve accuracy, we additionally employed the highly correlated DLPNO-CCSD(T) method for ionization energies and electron affinities in water. Finally, the electronic and global properties of biologically relevant protonated forms nicotinium, cotinium, caffeinium, theophyllinium, and theobrominium were investigated, providing a description of these compounds in aqueous solution. This approach enabled comparison of their structural, thermodynamic, electronic, and redox properties across the investigated molecular series. In addition to the implicit Conductor-like Polarizable Continuum Model (CPCM), an explicit solvation approach involving several water molecules was also tested for the nicotine-water aggregate.

## 1. Introduction

**Figure 1:**
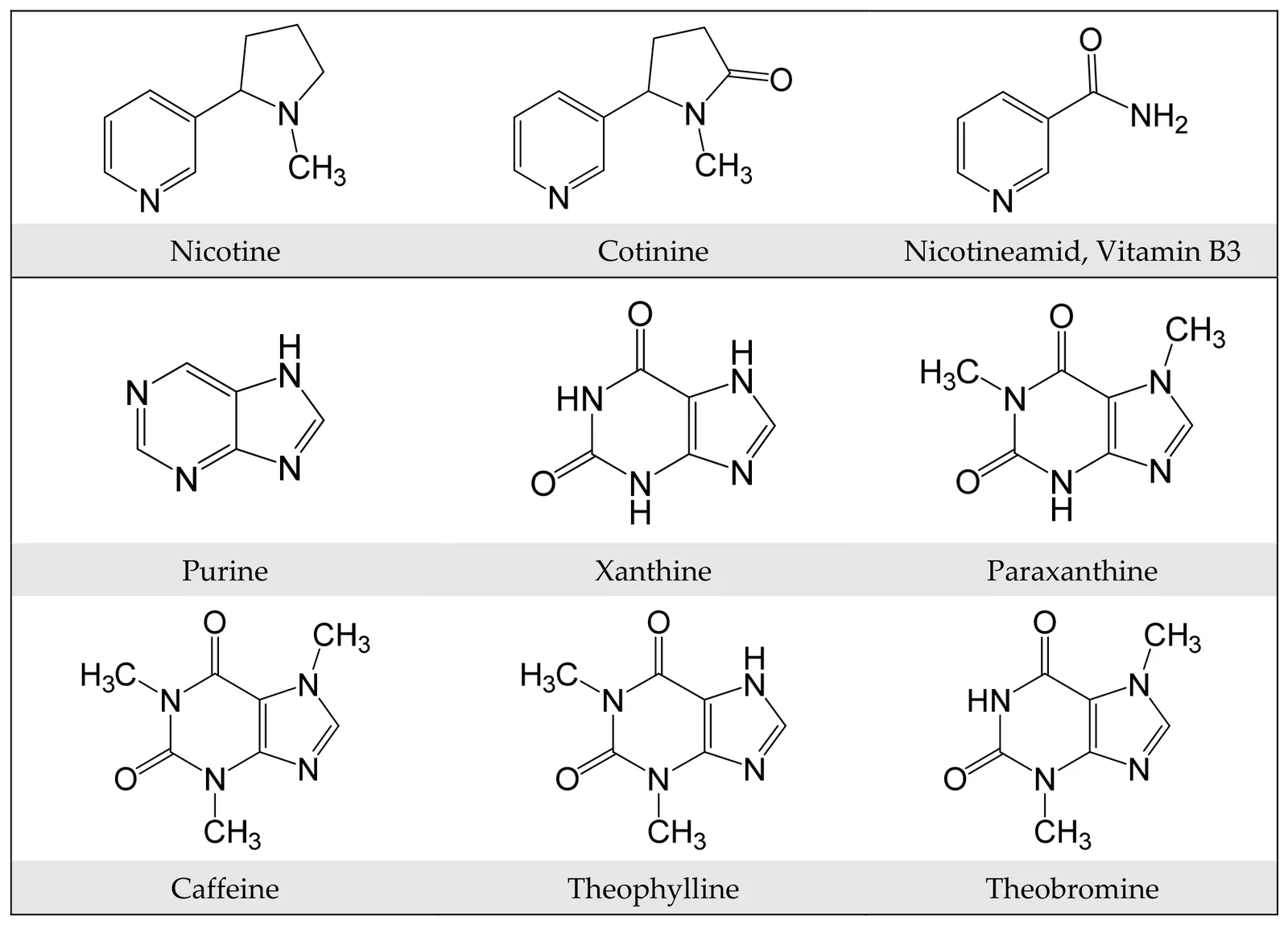
Some pyridine- and purine-type stimulants.

Stimulants (psychostimulants) are chemical substances that promote wakefulness. They are used to enhance attention, motivation, mood, cognition, and physical performance. They are either of natural or synthetic origin. They are classified as follows (Figure 1):

- Pyridine-type stimulants, including nicotine and cotinine;
- Purine-type stimulants, primarily including caffeine, xanthine, theophylline, and theobromine;
- Amphetamine-type stimulants (amphetamine, methamphetamine, ephedrine);
- Other stimulants, including, for example, cocaine, tetrahydrocannabinol (THC), and the hallucinogen lysergic acid diethylamide (LSD);
- Opiates, e.g., morphine, codeine, papaverine, and heroin.

Nicotine is a well-known and widespread drug contained in tobacco leaves. It is a natural alkaloid used as a recreational stimulant and possesses mild analgesic effects. It acts as a receptor agonist (activator) on nicotinic acetylcholine receptors and has a significant impact on the cardiovascular system (catecholamine release, increased heart rate and blood pressure); it is highly addictive. Nicotine has a half-life of 1–2 h. In the past, nicotine was used as an insecticide in the form of a tobacco extract sulphate. Neonicotinoids are neuro-active insecticides that are chemically similar to nicotine. Cotinine is an active metabolite of nicotine that remains in the blood, with a half-life of 18–20 h, which facilitates its analysis. Cotinine demonstrates antidepressant effects. Nicotinamide is the amide of nicotinic acid, also known as Vitamin B3.

Purine derivatives, which contain a fused imidazole and pyrimidine nucleus, are highly diverse. In addition to precursors of caffeine, they also include nucleic bases (adenine, guanine) as components of DNA and RNA. Purine is a water-soluble alkaloid found in a diverse range of foods. Caffeine is the most widespread drug, contained in coffee beans but also in other plants. It acts as a methylxanthine-type central nervous system (CNS) stimulant, where it blocks the binding of adenosine-to-adenosine receptors, thereby suppressing the inhibitory effect of adenosine and increasing the level of the neurotransmitter acetylcholine. Caffeine is found in coffee, tea, cola, and energy drinks; it is mildly addictive. It is added to analgesics. Caffeine is metabolized in the liver by demethylation to form paraxanthine, theobromine, and theophylline. Its half-life is 3–7 h. Theobromine is a dimethylxanthine and is an isomer of theophylline. It is primarily found in cocoa beans, from which it passes into cocoa butter and chocolate, lending them their characteristic bitter taste. Theophylline is also found in cocoa beans and is used in medicine (for asthma) and to enhance sporting performance. The physicochemical properties of these stimulants are detailed in Table 1. The core physicochemical properties of the studied stimulants were retrieved form public databases: PubChem https://pubchem.ncbi.nlm.nih.gov/compound/Name, accessed on 8 October 2026; Drugbank https://go.drugbank.com/drugs, accessed on 8 October 2026; HMDB (Human Metabolome Database), https://hmdb.ca/metabolites/, accessed on 8 October 2026; CCDC (Cambridge Crystallographic Data Centre) https://www.ccdc.cam.ac.uk/structures/, accessed on 8 October 2026.

**Figure 2:**
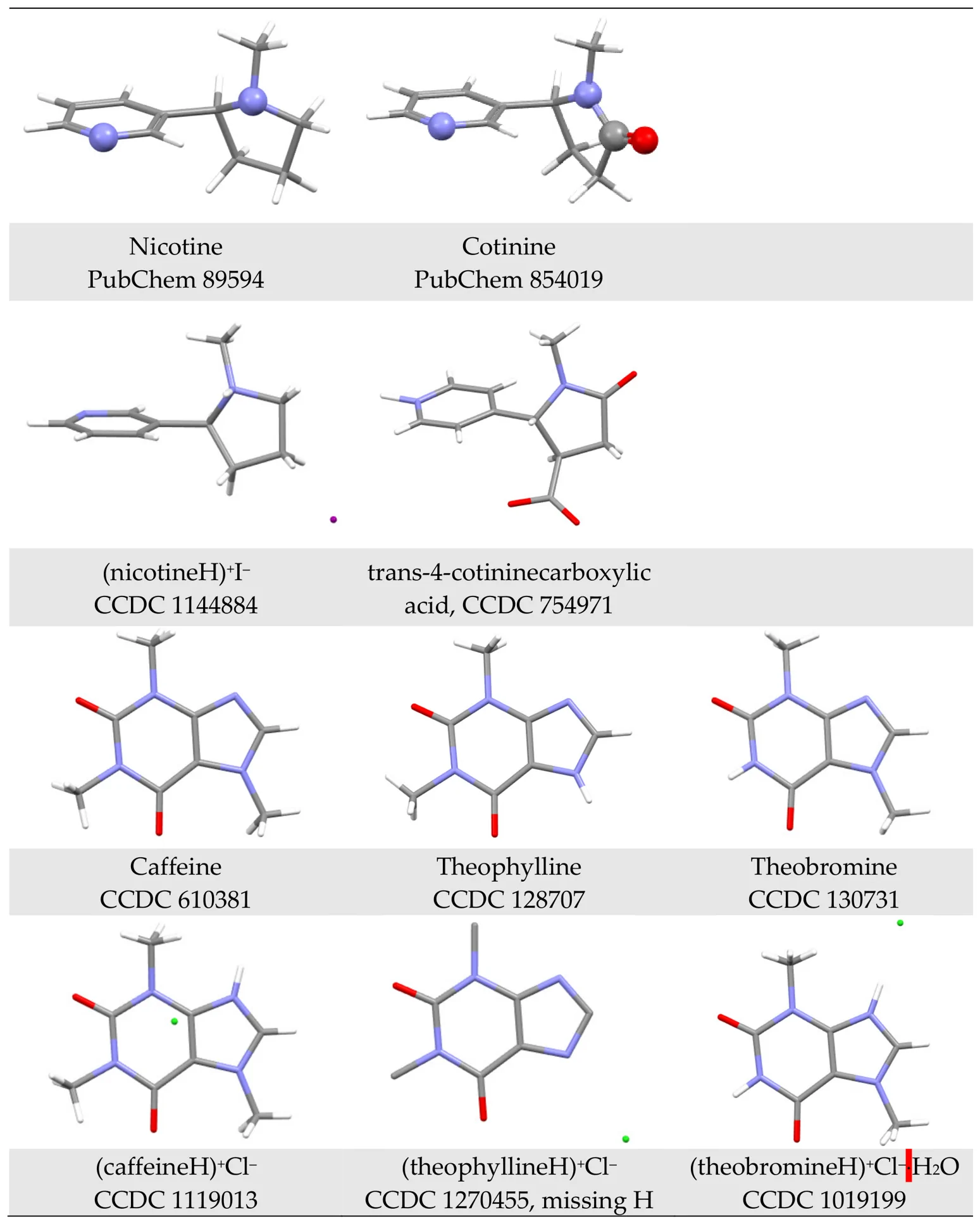
Structures of stimulants according to databases CCDC and PubChem (colour code: N, blue; O, red; H, white; C, grey; Cl, green; I, violet).

Nicotine and caffeine are highly soluble in water. Caffeine is very alkaline. The mentioned stimulants have a molecular structure which for the solid-state compounds and/or protonated salts were determined by X-ray diffraction and are deposited in the CCDC database (Figure 2).

The aim of this theoretical work is to calculate global reactivity indices and the absolute oxidation and reduction potentials of these vital compounds. This information helps elucidate the redox mechanisms of action of these species. Calculations were performed using the approximate B3LYP method based on density functional theory (DFT), including full geometry optimization and vibrational frequency analysis. In addition, the highly accurate ab initio DLPNO-CCSD(T) method was employed to account for the major part of the electron correlation energy.

## 2. Results

This section describes the molecular geometries of the psychostimulants (nicotine, cotinine, caffeine, theophylline, and theobromine) as determined using the B3LYP method for geometry optimization. These calculated structures were compared against available experimental (X-ray crystallography) and predicted data (PubChem, CCDC).

Both nicotine and cotinine are flexible molecules due to a rotatable C-C bond connecting the two rings, allowing them to exist as various rotamers (conformational isomers). Since nicotine is a liquid, an X-ray structure is unavailable. The starting structure was based on the X-ray data for the solid salt, nicotinium iodide (labeled as nicotine_1). A predicted structure from PubChem (labeled nicotine_2) was also available. The calculated geometry for nicotine (simulated in an aqueous environment) closely matches the reported solid-state structure of the nicotinium cation.

Cotinine is typically a viscous oil. The initial geometry was extracted from the CCDC structure of the related compound, trans-4-cotininecarboxylic acid (labeled cotinine_1). The PubChem database offered a structure for a different isomer, 3-cotinine (labeled cotinine_2). The final calculated geometries showed good agreement with both the CCDC and PubChem structural estimates.

The flexibility of the xanthine molecules (caffeine, theophylline, and theobromine) is limited primarily to the rotation of the terminal –CH_3_ groups attached to the ring system. The geometries calculated for these species nearly perfectly match the experimental data obtained from solid-state X-ray structures.

The calculated total molecular energies (*E^q^*) and Gibbs free energies (*G^q^*^,ø^) for the neutral (C^0^), oxidized (C^+^), and reduced (C^−^) canonical forms were compiled (Table S1). Protonation of these molecules leads to the formation of the -ium ions (e.g., nicotinium, caffeinium), which are stable in acidic solutions. The calculated molecular geometries for these protonated ions (P^+^) agree well with the solid-state structures reported for their corresponding hydrogen chloride salts.

**Molecular descriptors.** A set of ground-state molecular descriptors is presented in Table 2. The polarity of molecules is measured by the permanent dipole moment, which is rather high: *p* = 3.4 (nicotine)–6.4 (theobromine) debye. The dipole polarizability is also high: *α*/a_0_^3^ = 188 (nicotine) and 169 (theobromine). The zero-point vibrational energy varies between *E*_zpe_ = 141 (nicotine) and 100 kcal mol^−1^ (theobromine), while the total entropic term varies only slightly: *S*^ø^*T*^ø^ = 30–33 kcal mol^−1^.

**Figure 3:**
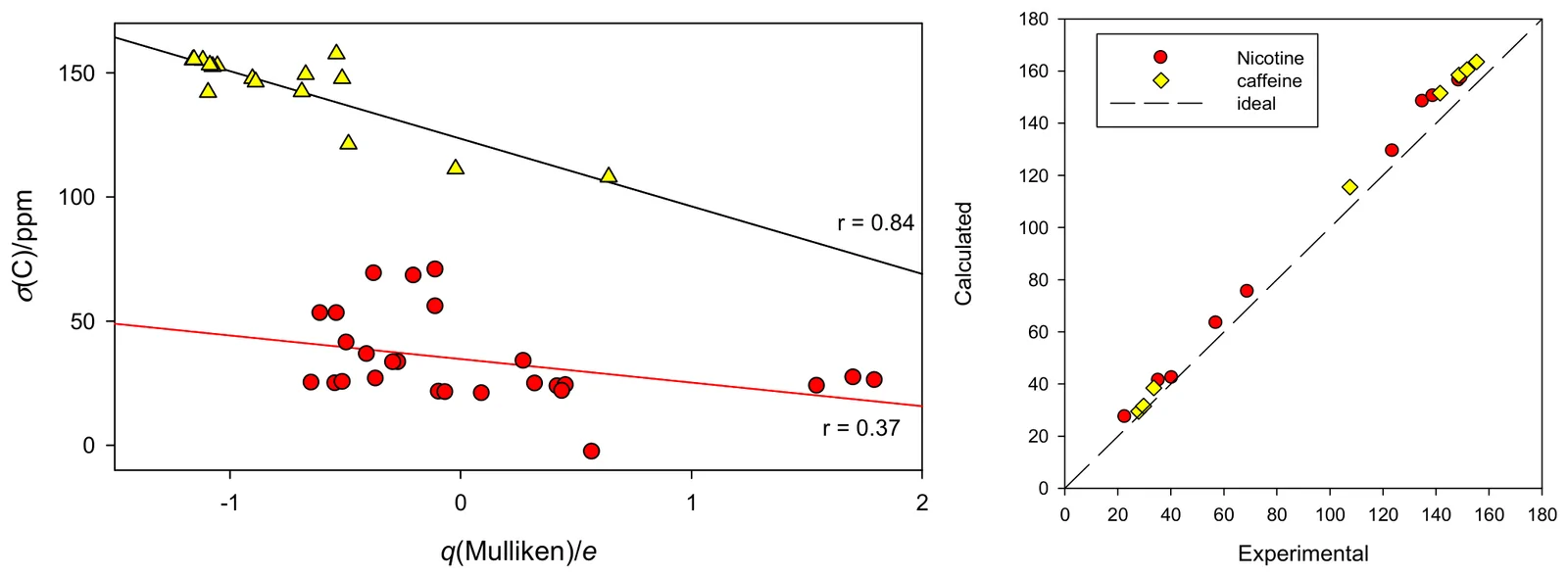
(**Left**): Correlation of absolute ^13^C NMR shielding constants with atomic (Mulliken) charges for stimulants: *σ*(C^2^_ring_)—circles, *σ*(C^3^_al_) —triangles; *r* is the correlation coefficient of the linear regression. (**Right**): Comparison of ^13^C NMR chemical shifts *δ* of nicotine and caffeine against TMS in ppm. Calculated B3LYP in water σ(C,TMS) = 184.5 ppm. Experimental data: https://pubchem.ncbi.nlm.nih.gov/compound/nicotine accessed on 8 October 2026, https://pubchem.ncbi.nlm.nih.gov/compound/caffeine accessed on 8 October 2026.

Calculated ^13^C NMR shielding constants clearly differentiate between lower aromatic σ(C^2^_ar_) and higher aliphatic σ(C^3^_al_) values (Table 3). The population analysis offered the Mulliken atomic charges, which were correlated with the carbon shielding constants (Figure 3). The correlation is rather weak, but the carbon atoms involved have very different bonding properties and environments. For example, the carbon atom in the five-membered non-aromatic ring of cotinine with a >C=O environment has a very low shielding constant σ(C^2^_al_) = –2 ppm and the Mulliken charge *q* = +0.57 *e*. In caffeine (and analogously in theophylline and theobromine), the most positive charge has the atom *q*(C^2^_ar_) = +1.75 *e* with σ(C^2^_ar_) = 26 ppm; this atom is common for both rings and is adjacent to the “naked” N^2^_ar_ with σ(N^2^_ar_) = 6 ppm.

Calculated absolute ^13^C NMR shielding constants were converted to the relative scale against the standard tetramethylsilane (TMS), and they were correlated with experimental data for nicotine (Figure 3 right). The calculated data are systematically overestimated, but a qualitative agreement is satisfactory.

**Global electronic properties.** The adiabatic ionization energies (AIEs) and electron affinities were evaluated after independent geometry optimization of the molecular cation and anion. Then, the molecular electronegativity, chemical hardness, and electrophilicity index were evaluated. The reaction Gibbs energies on ionization were converted to the absolute oxidation and reduction potentials (Table 4).

The literature data on ionization energies of stimulants involve some experimental data and calculated values; these refer to the vacuum state. For instance, the accurate adiabatic ionization energy of theophylline was determined as *E*_i_(**4**) = 8.184 eV = 188.7 kcal mol^−1^ and for theobromine *E*_i_(**5**) = 8.133 eV = 187.6 kcal mol^−1^ in vacuo [8]. In order to compare the quality of calculations, B3LYP (M1) and DLPNO-CCSD(T) (M2) methods were used in vacuo for the neutral and ionized molecule of theophylline, yielding *E*_i_ = 183.5 and 188.8 kcal mol^−1^ for M1 and M2, respectively. Thus, the DLPNO-CCSD(T) method, in accordance with expectations, recovers the ionization energies almost perfectly.

The methylxanthines (caffeine, theophylline, and theobromine) are structural analogues that differ in the position and number of methyl groups. Their ionization energies are relatively close (135–137 kcal mol^−1^ for B3LYP data/141–143 kcal mol^−1^ for DLPNO-CCSD(T) data in water), indicating that the highest occupied molecular orbital (HOMO) from which the electron is removed is related to the common xanthine ring structure, often involving the lone pair on the nitrogen atoms. Another set of adiabatic ionization energies calculated by B3LYP in vacuo was reported as 7.90 eV for caffeine, 8.06 eV for theophylline, and 8.10 eV for theobromine (182, 186, and 187 kcal mol^−1^) [9].

Nicotine and cotinine have different core structures. Nicotine’s first ionization step is generally attributed to the lone pair of the pyrrolidine-ring nitrogen, which makes it slightly easier to ionize than the xanthine derivatives. An experimental value for the ionization energy of nicotine determined by photoelectron spectroscopy in vacuo is approximately 8.05 or 8.01 eV (185 kcal mol^−1^) [10]. Recent reinvestigation reports a value of 8.33 eV (vertical ionization data) [11]. To our best knowledge, there is not an unambiguous report on the experimental adiabatic ionization of nicotine energy. Our calculated adiabatic ionization energy of nicotine_1 in vacuo was 169.0 kcal mol^−1^ (7.33 eV) by B3LYP and 177.0 kcal mol^−1^ (7.68 eV) by DLPNO-CCSD(T). For nicotine_2, *E*_i_ = 168.0/176.2 kcal mol^−1^ in vacuo.

**Figure 4:**
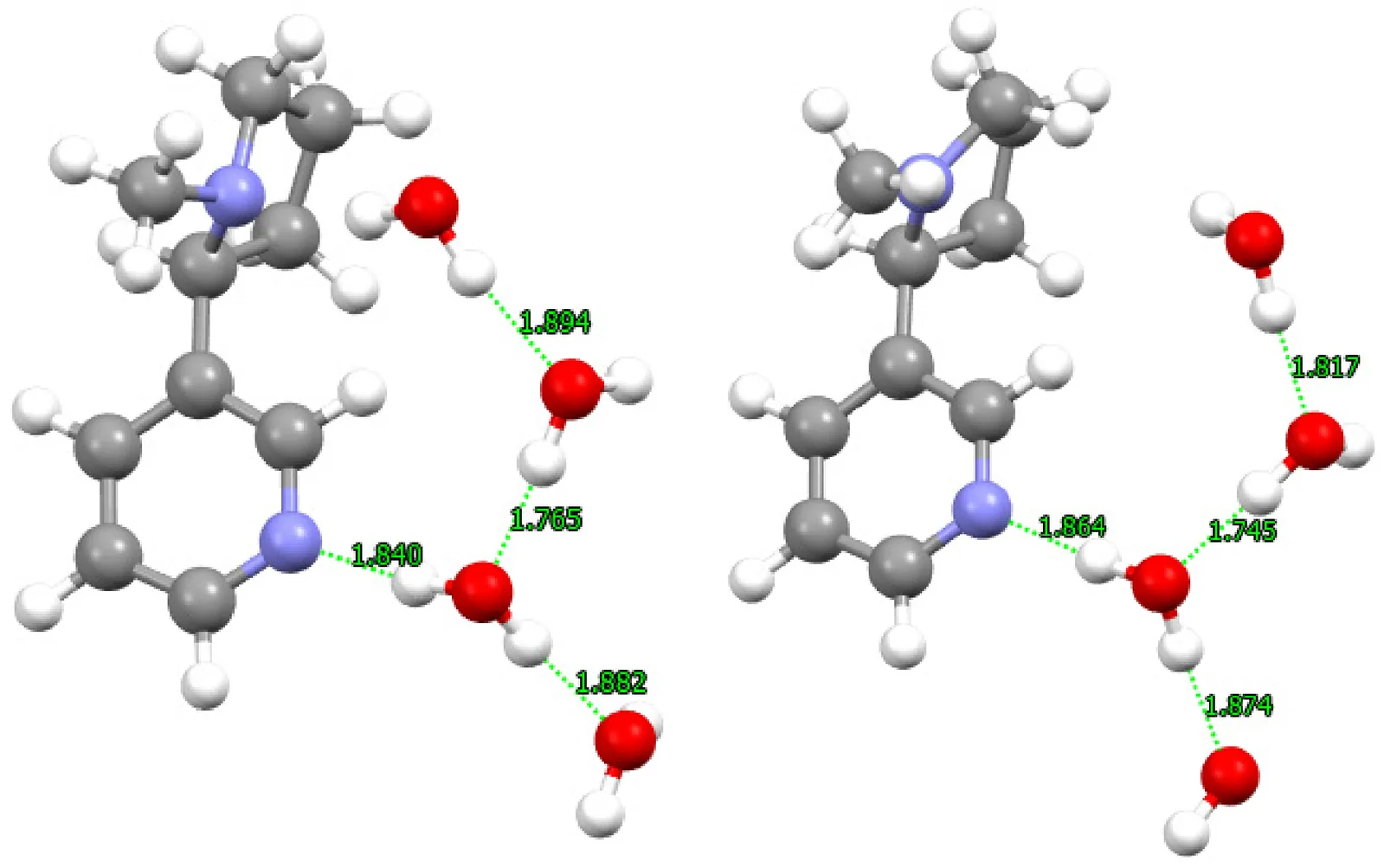
Optimized geometry of an aggregate nicotine·4H_2_O (**left**) and its cation (**right**), (colour code: N, blue; O, red; H, white; C, grey).

In order to go beyond the implicit solvation (CPCM) model, the explicit solvation with several water molecules was also modeled. The molecule of nicotine was surrounded by four water units in the hydrophilic region, and complete geometry optimization was performed. Then, two sorts of ionization energies were evaluated: (i) the vertical *E*_iv_ in the fixed geometry of a neutral molecule, and (ii) the adiabatic *E*_ia_ after geometry reoptimization of the molecular-aggregate cation. During the geometry optimization of the aggregate, the initially randomly oriented water molecules reposition themselves to form intermolecular hydrogen bonds (Figure 4). The calculated ionization energies for nicotine determined by various methods are listed in Table 5. It is seen that the explicit solvation resulted in a minor effect on the calculated ionization energies.

## 3. Discussion

Due to low ionization energy in aqueous environments (*E*_i_ = 118/125 kcal mol^−1^), nicotine can display a dual functionality: it can act like an antioxidant capable of scavenging free radicals. On the other hand, as a prooxidant, it can trigger the generation of excessive Reactive Oxygen Species (ROS), leading to cellular damage in various tissues. Cotinine has a higher AIE, suggesting oxidation makes it less prone to losing an electron: *E*_i_ = 150/157 kcal mol^−1^ in water.

A potential antioxidant capacity of caffeine was studied by the computational DFT method with the M05-2X functional and the 6-31+G(d)/6-311++G(d,p) basis set in water and benzene as solvents [12]. Radical adduct formation (RAF) has been identified as the main mechanism involved in the direct ROS scavenging activity of caffeine. Concurrent mechanisms assume the oxidation of caffeine (electron transfer) in two consecutive steps or one single step (SET—Single Electron Transfer, SEPT—Sequential Electron-Proton Transfer, PCET—Proton-Coupled Electron Transfer, and HAT—Hydrogen Atom Transfer mechanisms). As the ionization energy of caffeine in water is not small (141 kcal mol^−1^ by the DLPNO-CCSD(T) method), the single electron transfer is not supported. Typical antioxidants have *E*_i_ < 130 kcal mol^−1^ and even lower in deprotonated (anionic) forms.

**Figure 5:**
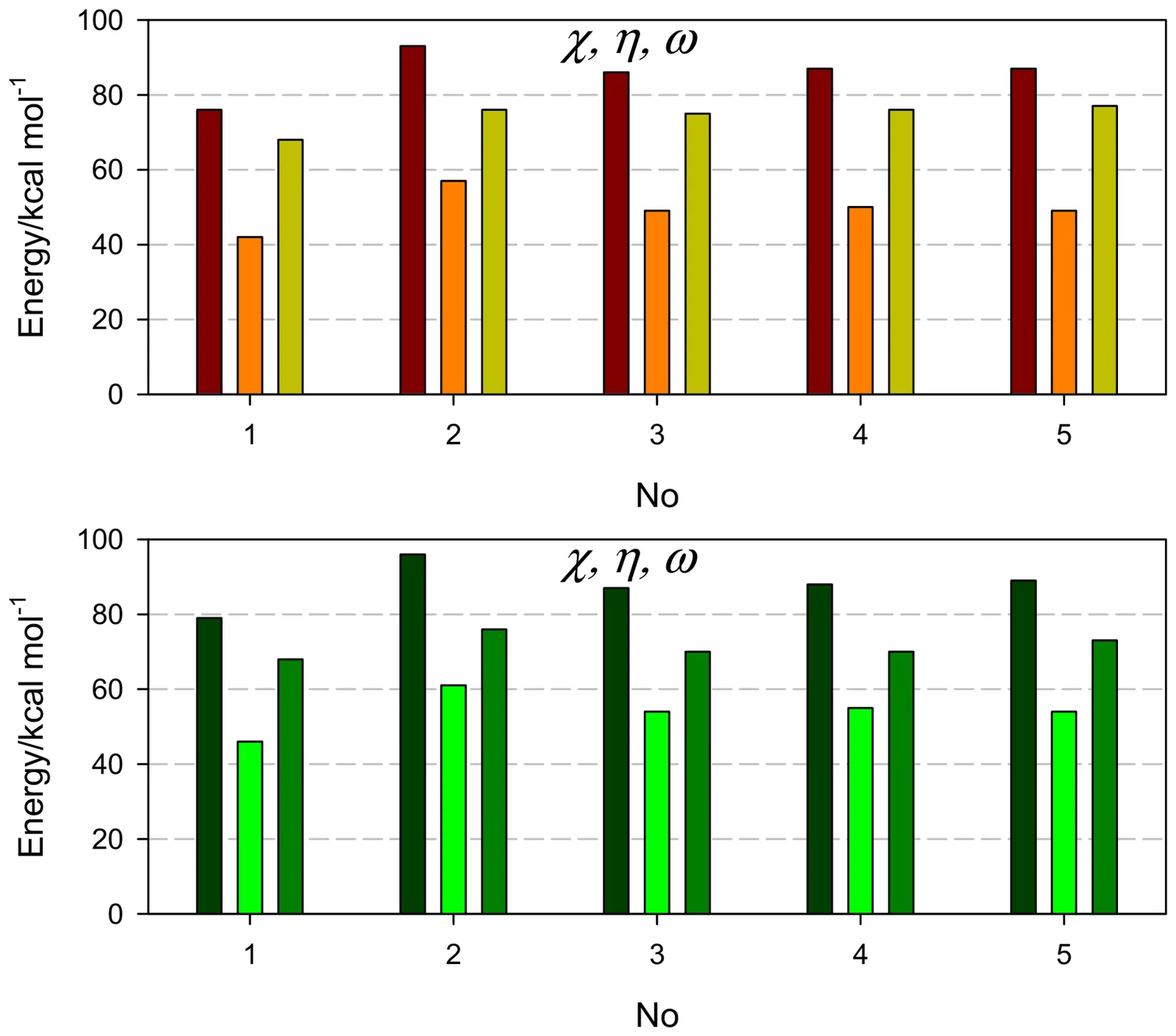
Comparison of global electronic properties of stimulants by B3LYP (**top**) and DLPNO-CCSD(T) (**bottom**). 1—nicotine, 2—cotinine, 3—caffeine, 4—theophylline, 5—theobromine; columns from the left: *χ*—molecular electronegativity (dark red/dark green), *η*—chemical hardness (orange/light green), *ω*—electrophilicity index (yellow/forest green).

Molecular electronegativity is the measure of a molecule’s propensity to attract and retain electrons. It quantifies the tendency of the electron cloud to shift towards the molecule. Chemical hardness measures a molecule’s resistance to change in its electronic charge or distribution. A system with high hardness is less polarizable and more stable. The electrophilicity index is a global measure of the propensity of a molecule to acquire an electronic charge (i.e., its ability to act as an electrophile). It combines both the capacity to attract electrons (electronegativity) and the resistance to electron flow (hardness). A higher *ω* value indicates a stronger electrophile, meaning the molecule is more eager to accept electrons and participate in reactions with nucleophiles (electron donors). These three global electronic properties are compared graphically in Figure 5. Data for *χ*, *η*, and *ω* produced by the DLPNO-CCSD(T) method are a bit higher compared to the B3LYP variant (due to higher ionization energies), but their trends are conserved. Cotinine displays the highest electronegativity and hardness.

Comparing caffeine (1,3,7-trimethylxanthine) and theobromine (3,7-dimethylxanthine) reveals how the removal of a single methyl group from the xanthine core dramatically alters their solid-state, thermodynamic, and solubility profiles. Caffeine is highly soluble in water, dissolving at roughly 2170 mg/100 cm^3^ at room temperature. Theobromine is sparingly soluble, with a much lower room-temperature solubility of roughly 0.33 mg/100 cm^3^; thus, theobromine is orders of magnitude less soluble in water than caffeine. The solubility is governed by the change in Gibbs energy, which involves several contributions: lattice enthalpy, hydration enthalpy, and the entropy of solution. A favorable (negative) Δ_soln_*G* requires either a sufficiently exothermic enthalpy of solution Δ_soln_*H* or a large entropy increase *T*Δ_soln_*S* to overcome a high lattice energy. In comparisons like caffeine vs. theobromine, caffeine’s lower crystal lattice energy allows water to easily pull its molecules into solution, whereas theobromine’s high lattice energy creates an energetic barrier that its hydration enthalpy cannot overcome, resulting in poor solubility. Thus, the distinct difference in solubility is not dictated by the molecular dipole moment or dipole polarizability in the solvent; it is determined by the difference in lattice energy (enthalpy).

Theobromine possesses a significantly tighter, more stable crystal lattice packing, driven by intermolecular N—H…O hydrogen bonding networks, made possible by its unsubstituted imidazole/pyrimidine ring position. This strong cohesion yields a very high melting point (357 °C), alongside a high sublimation tendency. Consequently, its crystal lattice energy is considerably higher, making it difficult for water molecules to break the solid apart. Caffeine is fully methylated at all three available ring-nitrogen positions, meaning it cannot form intermolecular N—H…O hydrogen bonds in the solid state. Its crystal packing is held together by weaker dipole–dipole and van der Waals interactions, resulting in a lower melting point (238 °C) and a weaker lattice energy that facilitates rapid dissolution.

Caffeine readily forms hydrates (such as caffeine monohydrate) under aqueous solid–liquid equilibrium conditions because water molecules easily insert into its crystal architecture. Theobromine is highly resistant to hydration; it does not easily form hydrates or solvates and typically remains strictly anhydrous even when immersed in water for extended periods.

When comparing polarity, theobromine exhibits a higher permanent dipole moment (*p* = 6.5 D in water) compared to caffeine (5.6 D), largely due to the polarized N–H bond asymmetry in its ring. Caffeine features a slightly higher isotropic polarizability (*α* = 187 vs. 170 *a*_0_^3^) and a larger molecular volume because of the extra protruding methyl group, altering how its electron cloud responds to aqueous solvent reaction fields.

## 4. Materials and Methods

The first task was the calculation of molecular geometry by an appropriate quantum-chemical method. For such a purpose, the hybrid variant B3LYP of the Density Functional Theory was used, as implemented in the ORCA software package (versions 5.0.4 and 6.0.1, developed by the ORCA team at the Max Planck Institute for Coal Research, Mülheim an der Ruhr, Germany, in collaboration with FAccTs GmbH, Cologne, Germany) [13,14,15]. This semi-quantitative method allows geometry optimization for molecules containing 30–50 atoms in a reasonable computing time; the basis set def2-TZVPD consisted of the triple-zeta-valence + polarization and diffuse functions [16,17]. In the optimum geometry with minimum energy, complete vibrational analysis was performed, assuming that no imaginary frequencies were indicated. Then, the vibrational partition function allowed the evaluation of the thermodynamic quantities, such as the internal energy, entropy, and Gibbs energy. For the ground state of the neutral molecules, the molecular descriptors were evaluated: dipole moment *p*, quadrupole moment *Q*, electric polarizability *α*, zero-point vibrational energy *E*_zpe_, and the total entropic term *S*^ø^*T*^ø^ at the standard conditions.

The neutral molecule was ionized by subtracting or attaching an electron, and for such open-shell systems the geometry was reoptimized. The molecular cation was used in evaluating the adiabatic ionization energy *E*_i_ = *E*^+^ − *E*^0^, and analogously, the molecular anion defined the adiabatic electron affinity, *E*_eg_ = *E*^−^ − *E*^0^. These data were further used to determine the molecular electronegativity *χ* = (*E*_i_ − *E*_eg_)/2, the chemical hardness *η* = (*E*_i_ + *E*_eg_)/2, and the electrophilicity index *ω* = *χ*^2^/2*η* [18,19,20]. Finally, the reaction Gibbs energy on oxidation defined the absolute oxidation potential for the one-electron reaction *E*_ox_^ø^ = −(*G*^+^ − *G*^0^)/*F,* and, analogously, the absolute reduction potential was *E*_red_^ø^ = −(*G*^−^ − *G*^0^)/*F*, where *F*—the Faraday constant.

To push the accuracy limit for ionization energies and electron affinities, the highly advanced ab initio method, DLPNO-CCSD(T) (Domain Localized Pair Natural Orbital—Coupled Cluster Singles, Doubles, and perturbative Triples) was used for the B3LYP-optimized geometries [21,22,23,24]. This computationally demanding method is a cost-effective route to the “gold standard” of quantum chemistry, capturing over 99.7% of the electron correlation energy compared to CCSD(T) [25]. The aug-cc-pVTZ basis set was used, as this is specifically suited for high-level correlation methods [26].

The calculations were done using the CPCM (Conductor-like Polarizable Continuum Model) method, which simulates situations in aqueous environments [27,28,29]. This was applied in each step of the geometry optimization. Singlet spin states were considered for electroneutral canonical structures and positively charged protonated species; otherwise spin doublets were considered with a UHF or UKS approach.

All calculations, including the CPCM model, atomic charges, and NMR shielding constants (GIAO version), were performed using the ORCA 6.1.0 package [14,15,30].

## 5. Conclusions

This theoretical work aims to explore the local and global electronic properties of common psychostimulants in an aqueous environment, focusing on structure–property relationships. Standard quantum-chemical methods that account for electron correlation energy were employed in this study.

**Structural Validation**. The structural and electronic properties of five major psychostimulants (nicotine, cotinine, caffeine, theophylline, and theobromine) were comprehensively investigated using Density Functional Theory (DFT) via the B3LYP functional with the implicit CPCM aqueous solvation model. The optimized molecular geometries exhibited exceptional agreement with solid-state X-ray diffraction structures from the CCDC database, confirming the reliability of the B3LYP/CPCM approach for modeling these compounds in solution.

**Global Descriptors and Polarities**. The studied molecules showed a diverse range of polarities and energetic properties, with permanent dipole moments spanning from *p* = 3.4 (nicotine) to 6.4 D (theobromine), and dipole polarizabilities *a* varying from 188 (nicotine) to 170 *a*_0_^3^ (theobromine). Zero-point vibrational energies (*E*_zpe_) ranged significantly from 141 kcal mol^−1^ (nicotine) to 100 kcal mol^−1^ (theobromine), whereas total entropic terms (*ST*^ø^) remained nearly constant at 30 to 33 kcal mol^−1^.

**NMR Analysis**. A weak correlation was identified between absolute ^13^C NMR shielding constants and atomic Mulliken charges, reflecting the varied chemical bonding environments across distinct molecular rings.

**High-Level Benchmarking**. High-level ab initio DLPNO-CCSD(T) single-point calculations successfully reproduced experimental gas-phase reference data (such as the adiabatic ionization energy of theophylline), confirming that incorporating advanced electron correlation methods effectively captures the energetics of these psychoactive compounds.

**Redox Implications for Nicotine and Cotinine**. Nicotine displayed the lowest adiabatic ionization energy in water (118 kcal mol^−1^ by B3LYP and 125 kcal mol^−1^ by DLPNO-CCSD(T)), highlighting its dual potential to act either as an antioxidant radical scavenger or as a prooxidant trigger for excessive reactive oxygen species (ROS). In contrast, cotinine exhibited a significantly higher ionization energy of 150 by B3LYP and 157 kcal mol^−1^ by DLPNO-CCSD(T)), alongside the highest molecular electronegativity (*χ* = 93/96 kcal mol^−1^) and chemical hardness (*η* = 57/61 kcal mol^−1^) in the series.

**Biological Relevance of Cotinine**. Cotinine’s elevated electronic stability, hardness, and electronegativity heavily restrict its susceptibility to single-electron oxidation, which is proposed as a key contributing factor to its notably prolonged half-life in biological systems compared to nicotine.

**Solubility of caffeine and theobromine.** Comparing caffeine and theobromine reveals that their dramatic difference in aqueous solubility is governed by crystal lattice energy rather than molecular dipole moments and dipole polarizabilities, as theobromine’s tighter N**–**H...O hydrogen-bonded lattice creates an insurmountable energetic barrier compared to caffeine’s weakly packed structure. Consequently, caffeine readily dissolves and forms hydrates, whereas theobromine remains highly resistant to hydration and orders of magnitude less soluble.

## Figures and Tables

**Table 1:** Physicochemical properties of some stimulants.

	Nicotine	Cotinine	Caffeine	Theophylline	Theobromine
IUPAC name	3-[(2S)-1-methyl pyrrolidin-2-yl]pyridine	(5*S*)-1-methyl-5-(3-pyridyl) pyrrolidin-2-one	1,3,7-trimethyl-3,7-dihydro-1*H*-purine-2,6-dione	1,3-dimethyl-7*H*-purine-2,6-dione	3,7-dimethyl-1*H*-purine-2,6-dione
Molecular formula	C_10_H_14_N_2_	C_10_H_12_N_2_O	C_8_H_10_N_4_O_2_	C_7_H_8_N_4_O_2_	C_7_H_8_N_4_O_2_
Molar mass	162.23	176.21	194.19	180.17	180.17
Rotatable bonds	1	1	0	0	0
Solubility in water ^a^	1 g/cm^3^	117 mg/cm^3^	2.17 g/cm^3^	7.36 mg/cm^3^	0.33 mg/cm^3^
log*P*	1.17	0.07	−0.07	−0.02	−0.78
Acidity p*K*_a_	8.5	4.79	14	8.81	9.9
Refractivity ^a^/m^3^ mol^−1^	49.7	49.3	49.8	44.9	44.9
Polarizability ^a^/A^3^	18.6	18.6	18.9	16.9	16.8
PubChem CID ^b^	89594	854019	2519	2153	5429
DrugBank DB	00184		00201	00277	01412
HMDB	0001934	0001046	0001847	0001889	0002825
CCDC			610381 1119028	128707	130731
	·HI, 1144884		·HCl, 1119013	·HCl, 1270455	·HCl, 1019199

^a^ Predicted by calculations. ^b^ Databases: PubChem https://pubchem.ncbi.nlm.nih.gov/compound/Name, accessed on 8 October 2026; Drugbank https://go.drugbank.com/drugs, accessed on 8 October 2026; HMDB (Human Metabolome Database), https://hmdb.ca/metabolites/, accessed on 8 October 2026; CCDC (Cambridge Crystallographic Data Centre) https://www.ccdc.cam.ac.uk/structures/, accessed on 8 October 2026. Structures according to References [1,2,3,4,5,6,7].

**Table 2:** Molecular descriptors in water by B3LYP/def2-TZVPD ^a^.

	Molecule	*p*	–*Q*	*α*	*S*	*V*	*E*_zpe_	*S* ^ø^ *T* ^ø^	*ν*_0_
Canonical forms									
**1**	Nicotine_1	3.400	54.9	188.5	774	1427	141.4	29.9	33
**1b**	Nicotine_2	4.489	54.0	188.4	773	1431	141.4	29.8	31
**2**	Cotinine_1	4.260	64.2	188.1	790	1468	130.0	30.9	41
**2b**	Cotinine_2	7.924	60.6	186.6	792	1480	130.0	31.0	28
**3**	Caffeine	5.621	60.2	187.0	782	1434	117.6	33.3	73
**4**	Theophylline	5.133	55.6	169.3	719	1313	100.4	30.5	79
**5**	Theobromine	6.452	56.7	169.9	732	1326	100.4	30.7	57
Protonated forms									
**1+**	Nicotinium_1	10.28	44.2	173.5	779	1460	151.4	30.0	36
**1b+**	Nicotinium_2	9.62	43.6	173.2	781	1450	151.4	30.1	26
**2+**	Cotininium_1	17.52	43.4	178.6	795	1481	138.9	31.0	40
**2b+**	Cotininium_2	14.63	45.7	176.3	797	1485	138.9	31.0	38
**3+**	Caffeinium	14.08	46.9	177.1	796	1480	126.1	32.9	58
**4+**	Theophyllinium	14.64	42.4	159.9	732	1333	108.9	30.6	69
**5+**	Theobrominium	13.88	44.7	159.9	737	1345	108.6	30.8	60

^a^ Energetic quantities are in units of kcal mol^−1^; 1 kcal mol^−1^ = 4.184 kJ mol^−1^. Dipole moment *p*/D (debye, D = 3.336 × 10^−30^ A m s); isotropic quadrupole moment *Q*/e*a*_0_^2^, isotropic dipole polarizability *α*/*a*_0_^3^, solvated surface *S*/*a*_0_^2^, solvated volume *V*/*a*_0_^3^ (bohr, *a*_0_ = 5.292 × 10^−11^ m); the lowest vibrational frequency (wavenumber) *ν*_0_ in cm^−1^, zero-point vibrational energy *E*_zpe_, total entropic term *S*^ø^*T*^ø^/kcal mol^−1^, standard temperature *T*^ø^ = 298.15 K.

**Table 3:** NMR parameters for stimulants in water by B3LYP/def2-TZVPD ^a^.

	Molecule	σ(H)	σ(C^2^_ar_)	σ(N^2^_ar_)	σ(C^3^_al_) or σ(C^2^_al_)	σ(N^3^_al_)	σ(O^2^)
**1**	Nicotine_1	22–30	27, 28, 34, 36, 55	−81	109, 142, 157, 121, 143	174	
**2**	Cotinine_1	22–30	18, 23, 25, 52, 57	−78	110, 154, 146, 150, -2(O)	108	−3
**3**	Caffeine	24–29	21, 24, 26, 33, 69	6, 71, 76, 112	146, 153, 155		−8, 21
**4**	Theophylline	22–29	21, 23, 27, 37, 70	2, 76, 87, 114	152, 155		−5, 20
**5**	Theobromine	24–29	22, 23, 24, 33, 68	5, 70, 80, 112	147, 152		−1, 40

^a^ Absolute values of the shielding constants are reported in ppm. (Experimental data on chemical shifts of ^13^C refer relative to the standard TMS—tetramethylsilane.) C^2^, N^2^, and O^2^ mean hybridization SP^2^; C^3^, N^3^—hybridization SP^3^; ar—aromatic ring, al—aliphatic ring or –CH_3_.

**Table 4:** Global electronic properties in water (kcal mol^−1^) ^a^.

	B3LYP	HOMO	LUMO	*E*_i_	*E*_eg_	*χ*	*η*	*ω*	*E*_ox_^ø^/V	*E*_red_^ø^/V
	def2-TZVPD	*KS*	*KS*							
	Canonical forms									
**1**	Nicotine_1	−140.2	−23.5	117.8	−33.5	76	42	68	−5.06	1.62
**1b**	Nicotine_2	−139.8	−21.5	117.7	−32.0	75	43	65	−5.06	1.56
**2**	Cotinine_1	−158.9	−25.6	150.2	−36.1	93	57	76	−6.42	1.73
**2b**	Cotinine_2	−157.6	−24.7	148.2	−34.8	91	57	74	−6.33	1.68
**3**	Caffeine	−143.3	−25.4	135.1	−36.4	86	49	75	−5.86	1.58
**4**	Theophylline	−145.3	−27.7	137.1	−36.9	87	50	76	−5.95	1.60
**5**	Theobromine	−145.8	−26.7	136.5	−38.0	87	49	77	−5.92	1.65
	Protonated forms									
**1+**	Nicotinium_1	−172.8	−31.4	159.5	−41.4	100	59	85	−6.84	1.97
**1b+**	Nicotinium_2	−172.9	−28.8	159.5	−39.3	99	60	82	−6.85	1.90
**2+**	Cotininium_1	−164.0	−57.3	155.1	−65.9	110	45	137	−6.64	3.04
**2b+**	Cotininium_2	−164.3	−58.0	154.2	−65.9	110	44	137	−6.60	3.04
**3+**	Caffeinium	−162.5	−42.4	155.5	−55.4	105	50	111	−6.72	2.56
**4+**	Theophyllinium	−163.4	−43.7	156.3	−57.6	107	49	116	−6.75	2.66
**5+**	Theobrominium	−163.7	−43.6	156.5	−56.3	106	50	113	−6.76	2.58
**DLPNO-CCSD(T)**										
	aug-cc-pVTZ	*HF*	*HF*	*E*_i_	*E*_eg_	*χ*	*η*	*ω*	*E*_ox_^*^/V	*E*_red_^*^/V
	Canonical forms									
**1**	Nicotine_1	−212.8	19.0	125.4	−33.3	79	46	68	−5.44	1.44
**1b**	Nicotine_2	−211.3	19.0	125.3	−31.8	79	47	66	−5.43	1.38
**2**	Cotinine_1	−219.5	18.8	157.1	−35.2	96	61	76	−6.81	1.53
**2b**	Cotinine_2	−214.9	18.5	156.4	−34.6	96	61	75	−6.78	1.50
**3**	Caffeine	−219.4	18.8	141.1	−32.7	87	54	70	−6.12	1.42
**4**	Theophylline	−201.4	18.3	143.0	−33.5	88	55	70	−6.20	1.45
**5**	Theobromine	−201.4	18.7	142.3	−35.0	89	54	73	−6.17	1.52
	Protonated forms									
**1+**	Nicotinium_1	−222.9	17.6	166.1	−40.6	103	63	85	−7.20	1.76
**1b+**	Nicotinium_2	−222.4	17.5	165.9	−38.4	102	64	82	−7.19	1.67
**2+**	Cotininium_1	−244.2	16.6	161.6	−65.5	114	48	134	−7.01	2.84
**2b+**	Cotininium_2	−239.2	16.7	160.8	−65.9	113	47	135	−6.97	2.86
**3+**	Caffeinium	−219.9	17.1	161.0	−51.4	106	55	103	−6.98	2.23
**4+**	Theophyllinium	−221.0	16.8	161.8	−34.5	98	64	76	−7.02	1.50
**5+**	Theobrominium	−221.5	17.1	162.2	−52.3	107	55	105	−7.03	2.27

^a^ Energetic quantities are in units of kcal mol^−1^; 1 kcal mol^−1^ = 4.184 kJ mol^−1^; 1 eV = 23.06054 kcal mol^−1^. Adiabatic ionization energy *E*_i_ = *E*^+^ − *E*^0^, electron affinity *E*_eg_ = *E*^−^ − *E*^0^, molecular electronegativity *χ* = (*E*_i_ − *E*_eg_)/2, chemical hardness *η* = (*E*_i_ + *E*_eg_)/2, electrophilicity index *ω* = *χ*^2^/2*η*. Absolute redox potential *E*_abs_^ø^(*L*^0^/*L^q^*) [V] = –Δ_react_*G*^ø^[J mol^−1^]/*F*, Faraday constant *F* = 96,485 A s mol^−1^; approximation *E*_ox_^*^ = –*E*_i_/*F*, *E*_red_^*^ = –*E*_eg_/*F*. HOMO—highest occupied molecular orbital, LUMO—lowest unoccupied molecular orbital, KS—Kohn–Sham orbitals, HF—Hartree–Fock orbitals.

**Table 5:** Various ionization energies (kcal mol^−1^) for nicotine in an aqueous environment by B3LYP/def2-TZVPD/CPCM(water).

Energy	Molecule, Nicotine	Aggregate, Nicotine·4H_2_O
*E*_iv_	136.2	145.2
*E*_ia_	117.7	120.7
Δ*G*^ø^	116.6	118.4

## Data Availability

The raw data supporting the conclusions of this article will be made available by the authors on request.

## Supplementary Materials

The following supporting information can be downloaded at https://www.mdpi.com/article/10.3390/molecules31203625/s1, Figure S1: Overlaid structures from CCDC or PubChem databases (green) with calculated optimized structures in water (red); Figure S2: Overlaid structures in vacuo (blue) and water (red); Table S1: Calculated energies in the optimum geometry of stimulants in water.

## Abbreviations

The following abbreviations are used in this manuscript:

B3LYP	Becke, 3-parameter, Lee–Yang–Parr
CCDC	Cambridge Crystallographic Data Centre
CNS	Central Nervous System
DFT	Density Functional Theory
DLPNO-CCSD(T)	Domain-Based Local Pair Natural Orbital Coupled Cluster with Singles, Doubles, and Perturbative Triples
DNA	Deoxyribonucleic Acid
HAT	Hydrogen Atom Transfer mechanisms
HF	Hartree–Fock Orbitals
HMDB	Human Metabolome Database
HOMO	Highest Occupied Molecular Orbital
KS	Kohn–Sham orbitals
LSD	Lysergic acid diethylamide
LUMO	Lowest Unoccupied Molecular Orbital
PCET	Proton-Coupled Electron Transfer
RAF	Radical Adduct Formation
RNA	Ribonucleic Acid
ROS	Reactive Oxygen Species
SEPT	Sequential Electron-Proton Transfer
SET	Single Electron Transfer
THC	Tetrahydrocannabinol
