# Supplementary material for: Electronic Properties of Common Psychostimulants in Aqueous Environments
Source: Molecules. 2026 Oct 12;31(20):3625. doi: 10.3390/molecules31203625 (PMC13707706; doi:10.3390/molecules31203625)
Supplement: Supplementary file 1 [file molecules-31-03625-s001.zip › molecules-4602653-supplementary.pdf]

Electronic properties of common psychostimulants in aqueous environments

Roman Boča, Beáta Vranovičová, Juraj Štofko, Cyril Rajnák
Faculty of Health Sciences, University of SS Cyril and Methodius, 91701 Trnava, Slovakia
Faculty of Natural Sciences, University of SS Cyril and Methodius, 91701 Trnava, Slovakia

Supporting Information

Table S1. Calculated energies in the optimum geometry of stimulants in water.[a]

B3LYP /def2-TZVPD		Canonical form, C			Protonated form, P		
Molecule/ion		C^+	C^0	C^-	P^{2+}	P^+	P^0
Nicotine_1-ccdc	E^q	-312918.87	-313036.71	-313070.26	-313167.63	-313327.18	-313368.54
	$G^{ø,q}$	-312801.29	-312917.92	-312955.37	-313040.56	-313198.37	-313243.91
Nicotine_2-pch	E^q	-312918.67	-313036.40	-313068.37	-313167.23	-313326.74	-313366.01
	$G^{ø,q}$	-312801.03	-312917.63	-312953.71	-313040.19	-313198.06	-313241.81
Cotinine_1-ccdc	E^q	-359364.90	-359515.09	-359551.16	-359644.98	-359800.04	-359865.96
	$G^{ø,q}$	-359260.23	-359408.28	-359448.23	-359531.20	-359684.34	-359754.51
Cotinine_2-pch	E^q	-359365.81	-359514.01	-359548.79	-359644.41	-359798.64	-359864.55
	$G^{ø,q}$	-359261.28	-359407.32	-359446.05	-359530.71	-359682.98	-359753.17
Caffeine	E^q	-426749.77	-426884.89	-426921.29	-427006.12	-427161.67	-427217.11
	$G^{ø,q}$	-426656.70	-426791.31	-426831.83	-426904.67	-427059.70	-427118.66
Theophylline	E^q	-402091.30	-402228.36	-402265.23	-402347.79	-402504.07	-402561.66
	$G^{ø,q}$	-402014.48	-402150.91	-402193.14	-402262.43	-402418.13	-402479.36
Theobromine	E^q	-402094.37	-402230.91	-402268.95	-402350.82	-402507.32	-402563.66
	$G^{ø,q}$	-402017.63	-402153.54	-402196.13	- 402265.77	-402421.72	-402481.33

DLPNO-CCSD(T) aug-cc-pVTZ		Canonical form, A			Protonated form, P		
Molecule/ion		C^+	C^0	C^-	P^{2+}	P^+	P^0
Nicotine_1	E^q	-312603.35	-312728.79	-312762.11	-312854.80	-313020.89	-313061.49
Nicotine_2	E^q	-312603.00	-312728.29	-312760.12	-312854.35	-313020.23	-313058.64
Cotinine_1	E^q	-358997.04	-359154.12	-359189.34	-359276.84	-359438.47	-359503.96
Cotinine_2	E^q	-358996.77	-359153.14	-359187.79	-359276.33	-359437.13	-359503.03
Caffeine	E^q	-426312.74	-426453.79	-426486.46	-426569.79	-426730.83	-426782.25
Theophylline	E^q	-401676.54	-401819.51	-401853.02	-401933.33	-402095.13	-402129.67
Theobromine	E^q	-401678.77	-401821.11	-401856.09	-401935.50	-402097.69	-402149.99

[a] Energy units are hartree (a.u.) = 627.5095 kcal mol^{-1}. q – molecular charge: -1,0,+1 for canonical forms C; +2,+1,0 for protonated forms P.

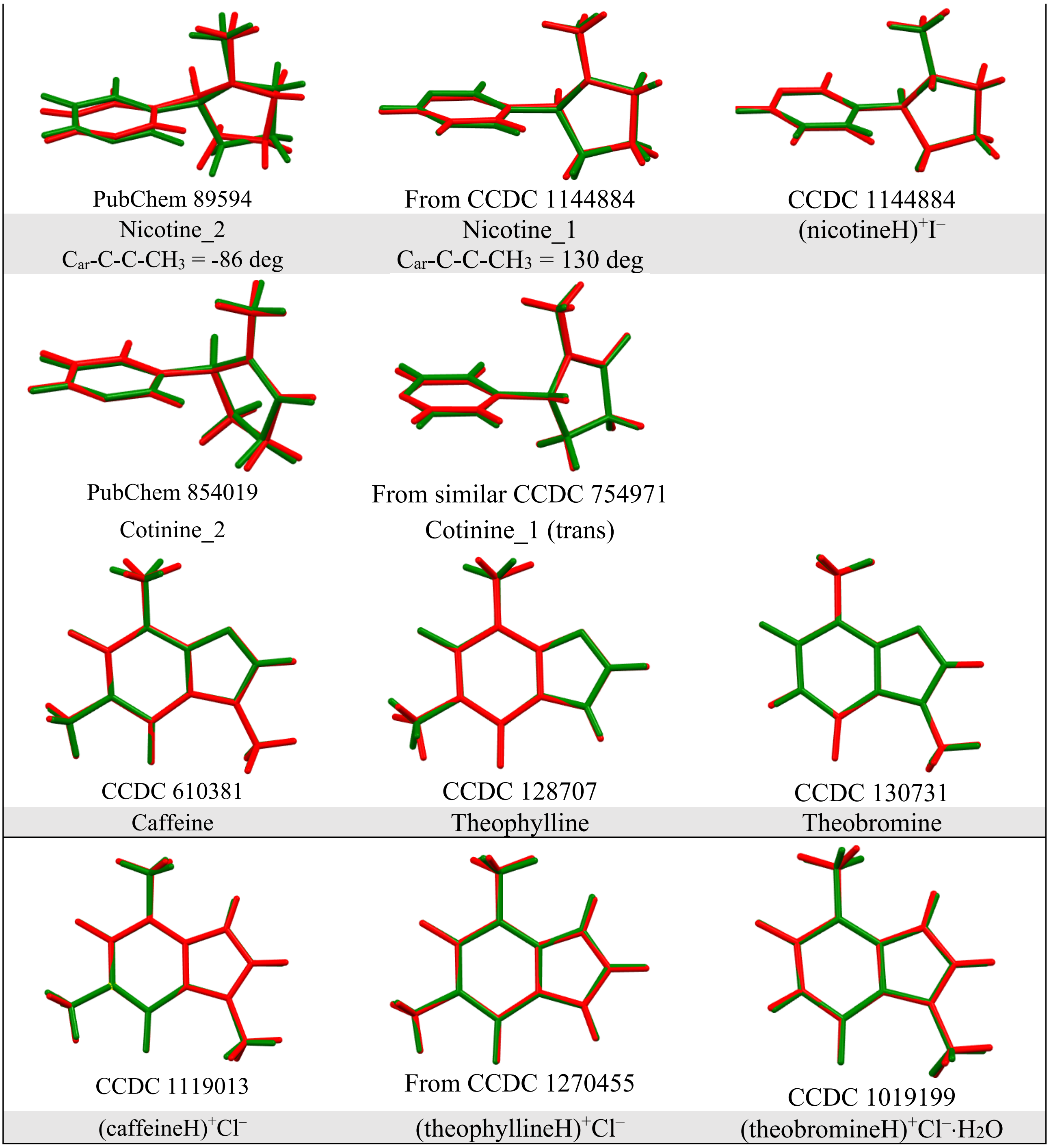

Figure S1. Overlaid structures from CCDC or PubChem databases (green) with calculated optimized structures in water (red).

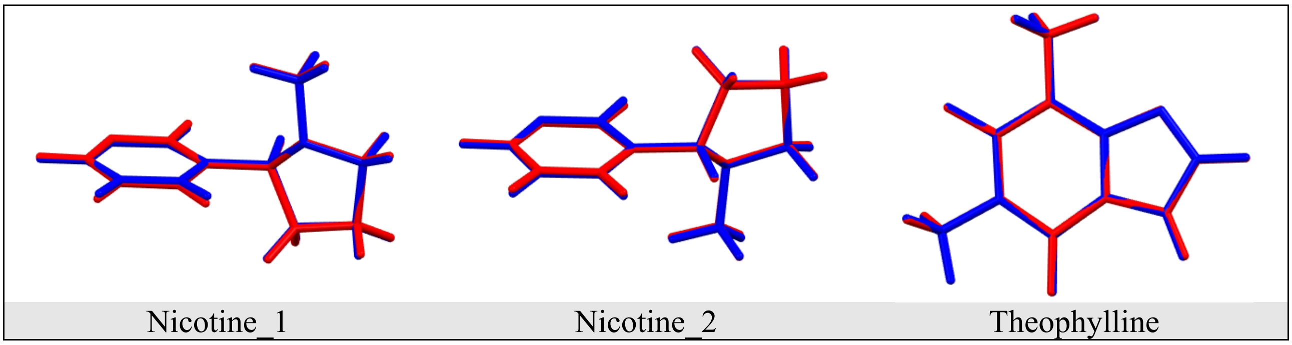

Figure S2. Overlaid structures in vacuo (blue) and water (red).
